# The effect of social interaction intervention on cognitive functions among non‐demented older adults: A systematic review and meta‐analysis

**DOI:** 10.1002/alz.084828

**Published:** 2025-01-09

**Authors:** Chi chuan Wei, Ming‐Jia Xie, Yi‐Fang Chuang

**Affiliations:** ^1^ Institute of Public Health, School of Medicine, National Yang Ming Chiao Tung University, Taipei, None Taiwan; ^2^ Taipei Veterans Hospital, Taipei Taiwan; ^3^ National Yang Ming Chiao Tung University, Taipei, NA Taiwan

## Abstract

**Background:**

Previous systemic reviews, predominantly including observational studies, have shown that participation in social activities is a protective factor against cognitive decline. However, this association is subject to potential reverse causality, creating a knowledge gap in our understanding of the effect of social interaction interventions on cognitive function. Therefore, this study aims to conduct a systematic review and meta‐analysis of randomized‐controlled trials to examine the effects of social interaction interventions on cognitive decline among non‐demented older adults.

**Method:**

This systematic review was registered in PROSPERO (CRD42022367828), and we systematically searched six databases from inception to May 6, 2022, to identify relevant articles on the effects of activities with social interaction components on cognitive function in community‐dwelling non‐demented older adults over 60. Two independent reviewers performed study selection, data extraction, and bias assessment, and RevMan5.3 was used to conduct the meta‐analysis. Subgroup analysis was conducted to explore potential sources of heterogeneity and assess variation in intervention effects across subgroups.

**Result:**

We included 11 studies for qualitative analysis and eight studies for the meta‐analysis. The result showed that social interaction intervention had a significant effect on executive function (SMD = 1.63; 95% CI, 0.55 to 2.70), but not attention (SMD = 0.09; 95% CI, ‐0.09 to 0.26) and memory (SMD = ‐0.03; 95% CI, ‐0.32 to 0.25). The subgroup analysis showed a greater cognitive benefit among normal older adults but not older adults with mild cognitive impairment. Additionally, when considering different social interaction modes, in‐person social interaction positively affected global cognition, while online interaction did not.

**Conclusion:**

Social interaction interventions have a limited impact on cognitive function in non‐demented older adults but showed potential effects on executive function. This finding offers insights for implementing social intervention in the community. Future efforts should focus on conducting more rigorous clinical trials to investigate the effects of different types of social interaction interventions on specific cognitive domains.

